# Fighting resistance: post-PARP inhibitor treatment strategies in ovarian cancer

**DOI:** 10.1177/17588359231157644

**Published:** 2023-03-01

**Authors:** Ana C. Veneziani, Clare Scott, Matthew J. Wakefield, Anna V. Tinker, Stephanie Lheureux

**Affiliations:** Division of Medical Oncology and Haematology, Princess Margaret Cancer Centre, Toronto, ON, Canada; Walter and Eliza Hall Institute of Medical Research, Parkville, VIC, Australia; Department of Medical Biology, University of Melbourne, Parkville, VIC, Australia; Royal Women’s Hospital, Parkville, VIC, Australia; Sir Peter MacCallum Department of Oncology, Peter MacCallum Cancer Centre, Melbourne, VIC, Australia; Walter and Eliza Hall Institute of Medical Research, Parkville, VIC, Australia; BC Cancer Agency, Medical Oncology Vancouver, Canada; Division of Medical Oncology and Haematology, Princess Margaret Cancer Centre, 610 University Ave, Toronto, ON M5B 2M9, Canada

**Keywords:** biomarkers, homologous recombination deficiency, ovarian cancer, PARP inhibitor, replication stress

## Abstract

Poly (ADP-ribose) polymerase inhibitors (PARPis) represent a therapeutic milestone in the management of epithelial ovarian cancer. The concept of ‘synthetic lethality’ is exploited by PARPi in tumors with defects in DNA repair pathways, particularly homologous recombination deficiency. The use of PARPis has been increasing since its approval as maintenance therapy, particularly in the first-line setting. Therefore, resistance to PARPi is an emerging issue in clinical practice. It brings an urgent need to elucidate and identify the mechanisms of PARPi resistance. Ongoing studies address this challenge and investigate potential therapeutic strategies to prevent, overcome, or re-sensitize tumor cells to PARPi. This review aims to summarize the mechanisms of resistance to PARPi, discuss emerging strategies to treat patients post-PARPi progression, and discuss potential biomarkers of resistance.

## Introduction

Epithelial ovarian cancer (EOC) continues to represent the second leading cause of gynecologic cancer death worldwide.^1^ High-grade serous ovarian cancer (HGSOC) is the most frequent histology, often diagnosed in advanced stages (III/IV).^2^ For the past two decades, the standard of care has been debulking surgery and systemic treatment with platinum-based chemotherapy in the first-line setting. Nevertheless, about 70% of patients with advanced disease relapse within the first 3 years.^3^ The first relapse is often sensitive to platinum; however, the disease becomes drug resistant over time. Since the potential synergy for targeting PARPi to *BRCA1* and *BRCA2* mutations was first reported in 2005, the treatment landscape of EOC has changed.^4^ Inhibition of PARP is synthetically lethal in EOC with *BRCA1/2* mutation and other aberrations leading to homologous recombination deficiency (HRD).^5^ The Cancer Genome Atlas has described that ~50% of HGSOC may harbor some form of HRD. In addition, HGSOC is characterized by *TP53* mutation, which plays an important role in the cell-cycle regulation. Therefore, HGSOC is susceptible to DNA-damaging drugs, such as platinum agents and PARPi.^6^

The integrity of double-stranded DNA is essential to maintaining genomic stability. DNA damage and its repair deficiencies have an essential role in HGSOC oncogenesis and their response to treatment. The DNA damage repair (DDR) system involves a complex molecular network of interconnected signaling pathways, with the main objective being the repair of DNA double-stranded breaks (DSBs) to maintain cell integrity.^7^ One of the most critical cell repair tools is homologous recombination (HR). HRD is a functional alteration whereby the DNA is unable to be repaired by HR. Consequently, cells employ alternative DSB repair by nonhomologous end joining (NHEJ), single-strand annealing, or microhomology-mediated end joining (MMEJ) pathways, which are error-prone DNA repair pathways causing high mutation rates.^8^ Unrepaired DSBs accumulate genomic aberrations resulting in instability which drives oncogenesis and can be detected as a ‘genomic scar’. These genomic changes are permanent and could represent mutations, insertions/deletions, and rearrangements. However, functional HR repair (HRR) can change over time (e.g. cells may regain the ability to repair DSBs) and drive mechanisms of drug resistance. HRD biomarkers include *BRCA1/2* and non-*BRCA1/2* HRD gene pathogenic or likely pathogenic variants and have become therapeutic targets, as well as predictive biomarkers.^9^

Poly (ADP-ribose) polymerase inhibitors (PARPis) have been approved in the first-line maintenance setting with unprecedented results in patients with EOC, especially in *BRCA*-deficient tumors. A recent analysis of long-term overall survival (OS) at 7 years in the SOLO-1 phase III trial (NCT01844986) confirmed the benefit of first-line maintenance olaparib in *BRCA*-mutated EOC. Olaparib compared to placebo led to clinically meaningful improvement in OS in patients with newly diagnosed advanced EOC harboring a *BRCA* mutation.^10^ The median OS was not reached in the olaparib group compared to 75.2 months in the placebo group (HR: 0.55, 95% CI: 0.40–0.76; *p* = 0.0004). This result is particularly remarkable as 44% crossover was reported, with data maturity of 38.1%. In the PAOLA-1 phase III trial (NCT02477644), no significant benefit in OS was observed with the addition of olaparib to bevacizumab compared to placebo and bevacizumab in first-line maintenance therapy in women with newly diagnosed advanced EOC. However, a significant improvement in median OS and progression-free survival (PFS) was observed in the HRD-positive subgroup. In this subgroup, the 5-year OS rates were 65.5% with olaparib plus bevacizumab *versus* 48.4% with bevacizumab and placebo (HR: 0.62, 95% CI: 0.45–0.85).^11^ The phase III PRIMA trial (NCT02655016) demonstrated benefit of niraparib in first-line maintenance irrespective of *BRCA* or HRD status.^12^ Updated data with 3.5 years follow-up revealed a sustained median PFS of 13.8 months with niraparib *versus* 8.2 months with placebo in all-comers (HR: 0.66; 95% CI: 0.56–0.79; *p* < 0.001).^13^ Recently, data from the phase III ATHENA-MONO trial (NCT03522246) also demonstrated that first-line maintenance rucaparib significantly improved median PFS in all-comers regardless of HRD status (HR: 0.52; 95% CI: 0.56–0.79; *p* < 0.0001).^14^

Despite impressive outcomes of PARPi, patients can present with de novo or, more frequently, acquired PARPi resistance. Studies of resistance mechanisms are paving the way for potential treatment opportunities targeting different resistance pathways. There is a need to characterize and define groups of patients who would benefit from PARPi rechallenge, combination therapy with PARPi, or alternative therapies. Retrospective data suggest that outcomes following PARPi are related to the prior response to platinum-based chemotherapy. Moubarak and colleagues analyzed 29 patients who had responded to the last platinum-based chemotherapy and were re-treated with PARPi. The study suggested a benefit in patients with unequivocal responses to the last platinum-based chemotherapy.^15^ Gadducci and colleagues analyzed the response to chemotherapy of 103 patients who received prior maintenance therapy with a PARPi and progressed. Better outcomes were seen in patients with a platinum-free interval (PFI) greater than 12 months.^16^

PARPis re-exposure was prospectively investigated for the first time in the phase IIIb OReO/ENGOT Ov-38 trial (NCT03106987). This study included women with recurrent platinum-sensitive EOC who must had have responded to their most recent platinum regimen and received a prior course of maintenance PARPi. Two cohorts were enrolled, a *BRCA*-mutation and a non-*BRCA*-mutation cohort, both of which were randomized to receive olaparib or placebo until disease progression. The majority of patients had at least three lines of chemotherapy, indicating a very sensitive selected population. Rechallenge with olaparib compared to placebo modestly prolonged median PFS compared to placebo both in *BRCA*-mutated (4.3 months *versus* 2.8 months, respectively, HR: 0.57; *p* = 0.022) or non-*BRCA*-mutated EOC (5.3 months *versus* 2.8 months, respectively, HR: 0.43; *p* = 0.002). Despite the statistically significant median PFS benefit, the Kaplan–Meier curve showed that about half of patients with a *BRCA-*mutated EOC rapidly progressed and had no benefit from PARPi rechallenge, despite responding to the prior platinum regimen. The exploratory analysis demonstrated a modest median PFS benefit of olaparib in the non-*BRCA*-mutation cohort, both in HRD-positive (median PFS 5.3 *versus* 2.8 months) and HRD-negative tumors (median PFS 5.4 *versus* 2.8 months). About 40% of tumors were HRD deficient. Patients with *BRCA* mutation who previously had less than 18 months of PARPi exposure and those without *BRCA* mutation who had less than 12 months of exposure failed to derive PFS improvement with olaparib.^17^

A recent post-hoc analysis of the SOLO2 trial (NCT01874353) used time to second progression to demonstrate reduced efficacy of platinum-based therapy lines following disease progression on maintenance olaparib in patients with *BRCA*-mutated platinum-sensitive EOC compared to patients who did not receive PARPi previously.^18^ Overall, these women achieved a longer time to first subsequent therapy and time to second subsequent therapy (TSST). The reported difference did not include the initial PARPi-derived benefit nor the total time to TSST.^18^ The ARIEL3 trial (NCT01968213) was designed to measure the impact of rucaparib as maintenance therapy in recurrent EOC after at least two lines of platinum chemotherapy. Of note, a high percentage of patients (45.8%) in the placebo group had crossed over to receive rucaparib. In all three cohorts – *BRCA* mutated, HRD positive, and intention to treat (ITT) – the median OS was similar with rucaparib and placebo (ITT: 36.0 *versus* 43.2 months, respectively; HR: 0.995, 95% CI: 0.809–1.223). The rucaparib arm demonstrated longer time-to-second disease progression (PFS2) among all cohorts.^19^

Regarding PARPi as treatment, the phase III ARIEL4 trial (NCT02855944) of rucaparib in relapsed *BRCA*-mutated EOC showed a decremental OS in the rucaparib arm, 19.4 months, compared to chemotherapy, 25.4 months (HR: 1.31, 95% CI: 1.00–1.73; *p* = 0.0507).^20,21^ This result may be driven by the OS result of the platinum-resistant subgroup (51% in the rucaparib arm and 49% in the placebo arm). Not only women with platinum-resistant EOC were less likely to benefit from PARPi, but those with *BRCA* reversion mutations did not benefit from rucaparib and subsequent therapies.^22^ Although platinum sensitivity and responsiveness are considered good clinical surrogate markers of PARPi response, the correlation is imperfect. For example, resistance to PARPi can occur in the context of the classical definition of platinum-sensitive disease by different mechanisms. To date, there is no established treatment-free interval defining sensitivity to PARPi.

Since PARPi therapies have moved to first-line maintenance, it is important to underscore that prior PARPi exposure will not be a synonym for PARPi resistance, and further characterization will be needed. Defining the mechanisms of PARPis resistance to develop treatment strategies is an urgent unmet need. This review aims to summarize the main mechanisms of PARPi resistance described and discuss potential strategies to prevent, overcome, or delay acquired resistance to these agents.

## DDR and PARP

The DDR system is essential to cell survival. PARP plays multiple roles in several DNA repair pathways, all of which could be involved in PARPi resistance. The DDR system involves a complex molecular network of interconnected signaling pathways, with the main objective being the maintenance of genomic integrity. Targeting this machinery is an emerging strategy to overcome PARPi resistance. There are six well-described DDR pathways: HR, NHEJ, base excision repair, nucleotide excision repair, Fanconi Anemia (FA) pathway, and mismatch repair (MMR).^23^ Alterations in any DDR pathways lead to genomic instability, a hallmark of cancer development. HR and NHEJ are two major pathways to repair DSBs.^24^ Recently, another DSBs repair mechanism named microhomology-mediated end joining (MMEJ) was described. MMEJ is associated with deletions alongside the break and contributes to translocations and rearrangements involving PARP1, DNA ligase III, and *POLQ* for the cell repair.^25^

A functional HR promotes accurate repair of DSBs, maintaining genomic integrity and cell survival. DNA damage sensors and signal transducers recruit DNA repair effectors to the sites of DNA breaks. Protein poly ADP-ribosylation (PARylation) is one of the first signaling steps upon sensing DNA breaks and initiates a cascade of protein recruitment to repair the DNA, which includes BRCA1 and the MRN complex (MRE11, RAD50, and NBS1).^26^ The MRN complex starts the 3′strand end-resection that is continued by other nucleases, such as *CTIP*, *DNA2*, and *EXO-1*. The pendent single-stranded DNA (ssDNA) is coated by replication protein A (RPA). BRCA1/2 and PALB2 facilitate RAD51 assembly onto ssDNA despite the high affinity with RPA. The generation of the RAD51-loaded filament is crucial for strand invasion of the sister chromatid and error-free DNA synthesis. Classical NHEJ is an alternative pathway predominant in HR-deficient cells for DNA repair. 53BP1 is a chromatin-binding protein that regulates the repair of DSBs and allows efficient NHEJ, particularly in BRCA1-deficient cells. This function requires interactions between 53BP1 and PTIP/RIF1.^27,28^ In G1 phase, the shieldin complex, which includes *REV7*, localizes the DSBs in a 53BP1 and RIF1-dependent manner.^29^ Shieldin protects against further end-resection diverting DNA repair toward the classical NHEJ. The Ku70/Ku80 complex bounds to the free ends of DNA, leading to the recruitment of DNA-dependent protein kinase catalytic subunits. PARP1 also binds to DNA ends in direct competition with Ku underlying the anti-NHEJ role of BRCA1 to counteract 53BP1 and control the levels of end-resected DNA.^29^

In all, 17 PARP proteins have been identified, of which PARP1, 2, and 3 have nuclear localization and are involved in DDR. PARP1 is the primary target of PARPi and is responsible for about 80–90% of the PARylation.^30^ PARP1 inhibition forces cancer cells to rely on error-prone DNA repair pathways or otherwise, unrepaired damage persists into mitosis, leading to the rapid accumulation of mutations, genomic instability, and eventual cell death in the context of HR deficiency.^31^ The primary mechanism of action of PARP inhibitors, as evidenced by the observed mechanisms of resistance, is trapping the PARP1 protein inducing replication fork collapse, and initiating replication fork protection. This trapping is mediated by steric factors, inhibition of the catabolic function, and interference with HPF1-modulated change in catalytic function.^32,33^ While combination therapies that reinforce this trapping and its downstream consequences will be the main approach, there may also be other combination opportunities that exploit the other diverse roles of PARP in biology.

**Figure 1.**
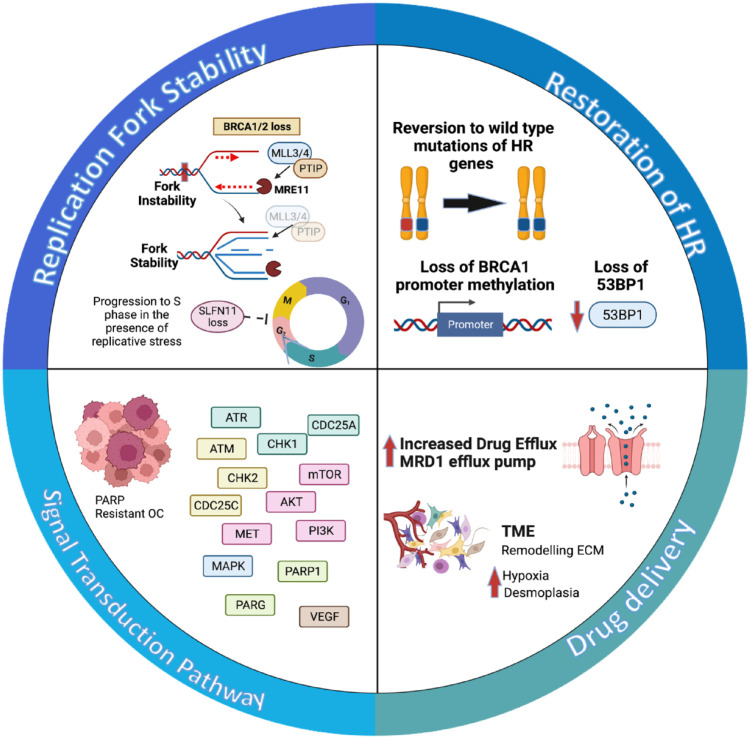
Mechanisms of resistance to PARPi. (a) The most common is the restoration of HR genes such as *BRCA* and *RAD51*. (b) Alterations in drug efflux pumps and the tumor microenvironment impair drug delivery leading to resistance. (c) Some signal transduction pathways promote HR and evasion of apoptosis. (d) Stabilization of replication fork allows DNA repair and fork restart. Created with BioRender.com. HR, homologous recombination; PARPi, poly (ADP-ribose) polymerase inhibitor.

DDR pathways are also controlled by kinases, such as ATM, ATR, and DNA-protein kinase, that initiate repair signaling cascades.^34^ Vulnerabilities in DDR pathways represent critical points of oncogenesis, opportunities to target novel therapeutics, and potential resistance mechanisms to genotoxic agents such as PARPi and platinum compounds.^35^ Among all the resistance mechanisms, the most studied are the restoration of HR, replication fork stability, alterations in drug delivery, and signaling transduction pathways (see Figure 1).

## Mechanisms of resistance

Given the use of PARPi in first-line therapy, the effort is not only directed to overcoming acquired resistance but to preventing it or re-sensitizing tumor cells to PARPi. Multiple mechanisms of PARPi resistance have been described and can be HR dependent or independent. HR-dependent mechanisms include reversion or secondary mutations in HRR genes,^36^ secondary mutations restoring *BRCA* function,^37^ and HR restoration by other alterations, such as mutations in Pax2 transactivation domain-interacting protein (*PTIP)*^38^ or *REV7*^39^ and loss of 53BP1 in *BRCA1*-deficient cells.^40^ Mechanisms that are HR-independent include loss of *PARG*,^41^ PARP activity alteration,^42^ and upregulation of drug efflux pumps.^43^

There are emerging techniques to identify mechanisms of PARPi resistance. CRISPR-based tools have provided significant insight into the mutational consequences of changes in PARP1, defining critical functional domains, and identified many of the genes we know can cause PARP inhibitor resistance.^44^ While these CRISPR screens have identified multiple genes involved in resistance, with many subsequently being validated in clinical cohorts, there has also been substantial variability between findings from published screens.^45^ These differences are likely heavily influenced by the genetic background the screen is undertaken in, which is often constrained by the experimental needs of such screens.

The specificity of mechanisms to background is illustrated by EZH2 loss only causing resistance in *BRCA2* mutants, and 53BP1 loss being involved in resistance in *BRCA1* mutations^40^ while increased activity of 53BP1 due to loss of KAT5 causes resistance in *BRCA2*-deficient cells.^46^ The impact of background is likely to be driven by the essential roles many of these proteins play in cell survival, with the limits of any adaptive change imposed by how far various steps in the pathway are perturbed by existing mutations and cell type expression patterns. These subtleties will continue to be elucidated as the number of diverse screens continues to grow, and CRISPR methods that alter expression rather than causing complete loss of function are employed. Careful validation in clinical cohorts and clinical discovery research guided by the mechanistic knowledge generated in these high-throughput screens will continue to play an important role in translating these insights into clinically meaningful strategies for fighting resistance.

In addition, many studies exploring PARP resistance mechanisms and synthetic lethality are ongoing (see Table 1), and there are still many unanswered questions. These strategies include combining therapies to amplify PARPi effects, targeting the acquired vulnerabilities, and delaying resistance by suppressing the mutator phenotype in HR-mutated tumors.^47^ The DDR-synthetic lethal concept is broader than PARPi, and the landscape of opportunity begins to be explored.

**Table 1.** Examples of active trials assessing different PARPi resistance pathways.

Study	Pathway	Treatment	Study population	Phase
NCT03162627	MEK/PARP	Selumetinib + olaparib	Recurrent OC with progression on PARPi	I
CAPRINCT03462342	ATR/PARP	AZD6738 + olaparib	Recurrent OC, platinum sensitive, HRD	II
EFFORTNCT03579316	WEE1/PARPATR/PARP	AdavosertibAdavosertib + olaparib	Recurrent OC, progressed on PARP	II
		Ceralasertib + olaparib		
NCT02723864	ATR/PARP	VX-970 + veliparib and cisplatin	Advanced refractory solid tumors including EOC	I
NCT03924245	HDAC/PARP	Entinostat + olaparib	Recurrent platinum refractory and resistant EOC	I/II
NCT02797977	CHK1	SRA737 + gemcitabine + cisplatin, or gemcitabine monotherapy	Advanced solid tumors, including HGSOC*BRCA*1/2 Wild type	I/II
NCT02915523	PD-L1/HDAC	Avelumab + entinostat	Advanced EOCWith progression after two lines of treatment	Ib/II
NCT04267939	ATR/PARP	BAY1895344 + niraparib	Advanced EOC with progression to PARPi treatment	Ib
NCT04586335	PI3K/PARP	CYH33 + olaparib	Advanced EOC with DDR gene mutations and/or PIK3CA mutations with progression on prior PARPi	I
NCT03579316	WEE-1/PARP	Adavosertib (AZD1775) +Olaparib, or adavosertib monotherapy	Recurrent EOC with progression on prior PARPi therapy	II
DUETTENCT04239014	ATR/PARP	Ceralasertib (AZD6738) +Olaparib, or olaparib monotherapy, or placebo	Relapsed platinum-sensitive OC, who have acquired resistance from prior PARPi treatment	II
TRESRNCT04497116	ATR / PARP	Camonsertib monotherapy or in combination with talazoparib or gemcitabine	Advanced solid tumors with ATR sensitizing mutations	I/IIa
ATTACCNCT04972110	ATR/PARP	Camonsertib + olaparib or niraparib	Advanced solid tumors resistant or refractory, molecularly selected	Ib/II
MYTHICNCT04855656	PKMYT1/ATR	RP-6306 + camonsertib	Advanced recurrent tumors with *CCNE1* amplification, FBXW7, and others	I
MAGNETICNCT05147272	PKMYT1	RP-6306 + gemcitabine	Advanced solid tumors with *CCNE1* amplification, FBXW7, and others	I
NCT04616534	ATR	Elimusertib + gemcitabine	Advanced EOC	I
MINOTAURNCT05147350	PKMYT1	RP-6306 + FOLFIRI	Advanced solid tumors with *CCNE1* amplification, FBXW7, and others	I

DDR, DNA damage repair; EOC, epithelial ovarian cancer; HGSOC, high-grade serous ovarian cancer; HRD, homologous recombination deficiency; OC, ovarian cancer; PARPi, poly (ADP-ribose) polymerase inhibitor.

### HRR-dependent mechanisms of PARPi resistance

#### Reversion mutations

Restoration of HRR function is a commonly acquired mechanism of platinum and PARPi resistance, occurring in about 20% of cases of EOC.^36^ In the SOLO3 trial (NCT02282020) of olaparib *versus* single-agent chemotherapy in patients with *BRCA* mutant, platinum-sensitive relapsed EOC, 22% of patients had secondary *BRCA* reversion mutations upon progression.^48^ These reversion mutations or re-expression of HRR-related genes that had been silenced through promoter hypermethylation may restore the HRR function. Reversion mutations restore the open reading frame of the *BRCA* gene, remove the original deleterious mutation, and restore the expression of a functional protein. Reversion mutations often show a microhomology signature, which suggests they resulted from the repair of DSBs *via* alternative error-prone repair mechanisms utilized in the setting of HR deficiency.^49^ Somatic reversions have been observed in other HR pathway genes such as *PALB2*, *RAD51C*, and *RAD51D* and are associated with poor prognosis.^50,51^ Furthermore, acquired loss of *RAD51C* promoter methylation leads to HR restoration and PARPi resistance.^52^ Translational analysis of ARIEL4 phase III trial assessing rucaparib *versus* chemotherapy (weekly paclitaxel *versus* platinum-based chemotherapy) in relapsed EOC with a *BRCA1* or *BRCA2* mutation identified fewer *BRCA* reversion mutations in three of four cases with platinum-resistant disease treated with weekly paclitaxel. This is an intriguing observation that needs to be further investigated.^20^

#### BRCA1 promoter alterations

*BRCA1* or *RAD51C* gene silencing through promoter methylation has been detected in ovarian and breast tumors.^53^ A potential mechanism of resistance to PARPi in these tumors involves gene re-expression. Pre- and post-platinum progression paired biopsies of EOC have shown that the de-silencing of *BRCA1* is linked to platinum resistance.^54^ As demonstrated in patient-derived xenograft (PDX) models of PARPi-resistant breast cancer, the loss of *BRCA1* promoter methylation restores functional *BRCA1* expression to the levels found in HR-proficient tumors.^42^ In EOC, susceptibility to PARPi was subsequently found to require *BRCA1* silencing by homozygous methylation of all copies present in the gene.^55^ Loss of promoter methylation of even one copy of *BRCA1* (heterozygous methylation) resulted in PARPi resistance.^42^

#### Restoration of end-resection

Other ways of regaining HRR proficiency without affecting the *BRCA1*-mutated status of the cell have been described, particularly in *BRCA1*-mutated cancer cells. Inactivation of the *TP53BP1* gene, which encodes the 53BP1 protein, is the most studied mechanism. Suppression of genomic instability is caused by *BRCA1*-mutated cells in the absence of 53BP1.^56^ Loss of 53BP1 activates ATM-dependent processing of broken DNA ends to produce recombinogenic ssDNA competent for HR.^40^ 53BP1 seems to act as the central component of a protein complex known as 53BP1–Shieldin to generate resistance to PARPi in *BRCA1-*mutated setting.^57^ The shieldin complex (SHLD1/2) promotes NHEJ by serving as the downstream effector of 53BP1, RIF1, and REV7, to counteract DSBs end-resection and promote DNA repair in *BRCA*-deficient cells.^58^ Loss of components of this complex results in the restoration of RAD51 foci formation and the ability to perform HRR in the absence of BRCA1.^59^ The depletion of REV7 was found *in vitro* to restore HR through *CTIP*-mediated end-resection, leading to PARPi resistance.^39^ Whole-genome CRISPR–Cas9 synthetic-viability/resistance screens in human *BRCA1*-deficient breast cancer cells treated with PARP inhibitors identified reduced SHLD1/2 expression in cells with intrinsic or acquired PARPi resistance.^60^ *DYNLL1* gene is a negative regulator of DNA end-resection by suppressing several components of the end-resection machinery, such as the MRN complex, in *BRCA1*-deficient EOC cells.^61^ *In vitro*, concurrent decrease in DYNLL1 expression in BRCA1-deficient cells resulted in PARPi resistance.^62^

In the *BRCA2* deficiency setting, preclinical findings of genome-wide CRISPR screen in *BRCA2*-knockout HeLa cell lines suggested that increased 53BP1 binding near the DSBs leads to reduction in end-resection and subsequent PARPi resistance due to an increase in NHEJ.^46^ These differences in mechanisms between *BRCA1* and *BRCA2*-deficient cells potentially reflect the different steps in HR-dependent DSBs repair in which each BRCA protein acts. However, further studies are warranted to verify these differences. Other mechanisms, such as decreased proteasomal degradation^63^ and amplification of wild-type *BRCA*, are involved in restoring HR capacity, also leading to PARPi resistance.^64^

### HRR-independent mechanisms of resistance

#### Restoration of replication fork stability

Replication stress results from the extremely increased cell growth and division in many tumors. Upon replication stress, replication forks stall and prolong cell-cycle arrest allowing time for DNA repair and re-entry into the cell cycle.^65^ Damage to replication fork protection evokes genomic instability and tumorigenesis.^66^ Treatment resistance promotes cancer cell progression through the cell cycle in the presence of DNA damage and replication stress; these include alterations in *RAD51*,^67^ *PTIP*, Chromodomain Helicase DNA Binding Protein 4 (*CHD4*), and the FA repair pathway.^65^ Downregulation of these factors in *BRCA1/2*-deficient cells leads to forkhead protection and PARPi resistance. *BRCA1/2* binds to stalled replication forks and protects them against degradation by the action of DNA nucleases. Deficiency in recruiting these nucleases to stalled replication forks or defective remodeling of the forks leads to PARPi resistance in *BRCA*-deficient cells. Fork degradation by MRE11 in *BRCA1/2*-deficient cells is promoted by the *PTIP*, *CHD4*, and *RAD52*. Inactivation of these protective factors and MRE11 inhibition protects DNA strands from extensive degradation.^68^ Similarly, the recruitment of the nuclease MUS81 by EZH2-directed histone methylation facilitates fork restart in *BRCA2*-deficient cells. Low EZH2 levels reduce MUS81 recruitment, lead to fork stabilization, and confer PARPi resistance only in *BRCA2*-deficient cells.^69^

#### SLFN11 loss

Loss of expression of the Schlafen 11 (*SLFN11*) gene is a common feature of human cancer cell lines and provides resistance to DNA-damaging agents, including PARPi.^70^ *SLFN11* is an executioner of replication stress, as it binds to RPA-coated ssDNA and blocks replication leading to cell death. Tumors with low expression of *SLFN11* depend on the ATR-CHK1-WEE1 axis to tolerate replication stress, and inhibition of this pathway can re-sensitize tumor cells to PARPi.^71^ *SLFN11* downregulation has been recently reported in disease progression on PARPi in two patients with EOC.^51^

#### Reduced cellular availability of PARPi

*ABCB1* encodes multidrug resistance protein 1 (MDR1), an ATP-binding cassette member involved in the cellular efflux of chemotherapeutic drugs. Overexpression of *ABCB1 via* fusions and translocations has been reported as a mechanism of acquired resistance to PARPi in EOC. A study reported 59% of fusions in specimens of recurrent HGSOC with the highest MDR1 expression.^72^ Most PARPi are MDR1 substrates, and particularly prior treatment with paclitaxel may induce MDR1 upregulation and indirectly induce PARPi resistance.^73^ Clinical findings with MDR1 inhibitors in drug resistance settings were disappointing.^74,75^ The addition of MDR1 inhibitors to PARPi in patients with *ABCB1* mutations has not yet been explored in clinical trials.^76^ In addition, other factors are responsible for PARPi bioavailability in the tumor cells. For instance, the tumor microenvironment impacts drug delivery leading to resistance. Various mechanisms are involved, including hypoxia, low PH, vascular abnormalities, shifts and polarizations in the immune cell population, and diverse stroma cells-derived secretomes, exosomes, and soluble factors. Hypoxia also regulates the microenvironment through the secretion of diverse cytokines.^77^

#### Poly(ADP-ribose) (PAR) glycohydrolase loss

The poly(ADP-ribose) (PAR) glycohydrolase (PARG) enzyme loss restores downstream PARP1 signaling upon PARPi treatment, counteracting synthetic lethality.^41^ Loss of PARG expression allows some PARylation to occur even in the presence of PARPi. This includes PARP1 auto-PARylation, which is an important event to allow PARP1 release from DNA. Consequently, PARG deficiency reduces PARP1 trapping and DNA damage accumulation. EOC cells that become PARPi resistant through PARG downregulation exhibit high replication stress and dependence on the ATR-CHK1-WEE1 pathway for survival. Thus, PARG downregulation confers sensitivity to CHK1 and WEE1 inhibition.^78,79^

#### Signal transduction pathway

Deregulation of multiple signaling pathways has been reported to be associated with PARPi resistance. The kinase c-MET phosphorylates PARP1 leading to its activation and reducing the binding affinity of PARPi, which results in PARPi resistance.^80^ Another mechanism is the upregulation of the ATM/ATR pathway, an essential checkpoint of the DNA damage response process due to its capacity to recruit DNA repair complexes through the phosphorylation of histone H2A. This phenomenon leads to HR restoration; thus, inhibiting this pathway is a strategy to overcome resistance.^81^ In addition, PARPi treatment upregulates the PI3K/AKT pro-survival pathway, which regulates cell growth and proliferation.^82^

## Strategies to prevent or overcome PARPi resistance

**Figure 2.**
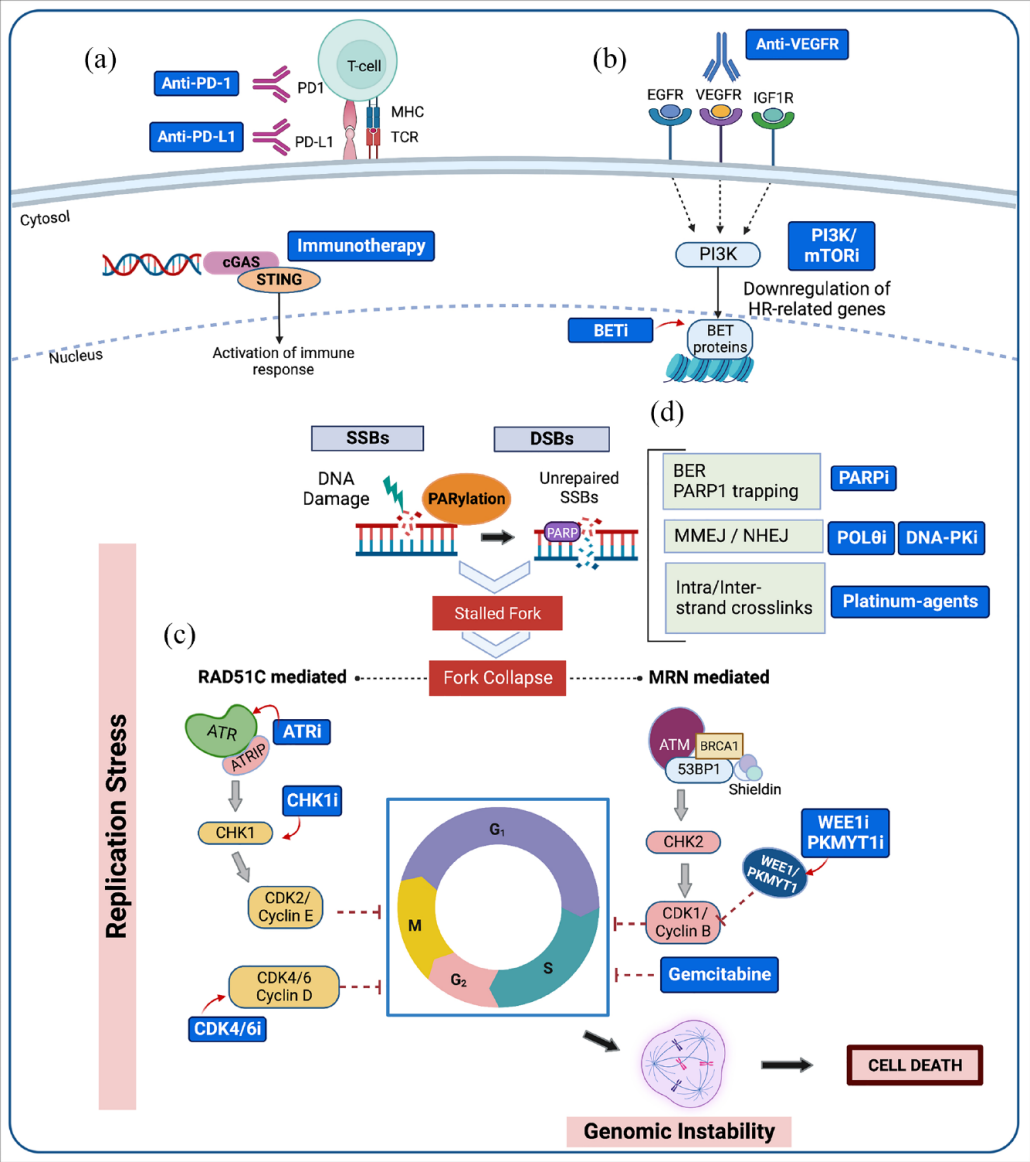
Overcoming resistance to PARP inhibitors. Various drug (blue boxes) combination strategies have been suggested to overcome or prevent PARPi resistance, promoting replication stress, genomic instability, and cell death. (a) Immune checkpoint inhibitors, such as anti-PD-1/PD-L1, might be an alternative approach given that HR-deficient tumors usually have high levels of genomic instability and are thought to present an increased number of neoantigens on their surfaces. (b) Reactivation of the HR pathway in tumors with acquired resistance to PARP inhibitors might be counteracted by various tyrosine kinase inhibitors (such as VEGF-targeted therapies) or agents targeting epigenetic regulators of HR-related genes (such as BET domain inhibitors). (c) Cancer cells depend on replication stress response for survival. This vulnerability can be targeted by inhibiting kinases (e.g. ATR, CHK1, WEE1, PKMYT1) that coordinate the DDR with cell-cycle control. (d) PARPis impair fork progression through PARP1 trapping and inhibition of the BER. POLQ inhibitors and DNA-PK inhibitors directly inhibit MMEJ and NHEJ. Platinum agents cause inter- and intra-strand crosslinks which increase DNA damage and impair fork progression. Created with BioRender.com. BER, base excision repair; BET, bromodomain and extra-terminal; DDR, DNA damage repair; HR, homologous recombination; PARP, poly (ADP-ribose) polymerase inhibitor; PD-1, programmed cell death protein 1; PD-L1, programmed cell death ligand 1; MMEJ, microhomology-mediated end joining; NHEJ, non-homologous end joining.

Many combinatorial strategies to overcome PARPi resistance are currently under development in preclinical or clinical settings (see Figure 2), such as PARPis combined with different anticancer agents. In this case, the combination might induce different mechanisms of actions of PARPis and contributes to the synergistic activity with each particular agent.^83^ Newer DDR-targeting agents (e.g. ATR, CHK1, WEE1, and PKMYT1 inhibitors) have emerged as a proposed combinatorial strategy to bypass PARPi resistance. These agents often carry a higher toxicity burden, usually manageable with dose adjustments and supportive measures.^84^ In general, inhibitors of ATR-CHK1-WEE1 are characterized by hematological toxicity, with anemia more prominent with ATR inhibitors and neutropenia with CHK1 and WEE1 inhibitors. However, safety data are still accumulating. Many combinations are thus far in early-phase stages, and additive toxicities and ideal doses are not robustly evaluated.

### Targeting molecular vulnerabilities

Abrogation of cell-cycle checkpoint signaling mitigates resistance to PARPi. Multiple inhibitors of cell-cycle checkpoint kinases such as ATR, ATM, CHK1, PKMYT1, and WEE1 are being investigated in EOC to circumvent PARPi resistance.^85^ CHK1 mediates cell-cycle arrest in the S-G2 phase and limits mitotic entry through phosphorylation of cyclin-dependent kinase 1 (CDK1). CDK1 is negatively regulated by WEE1 and PKMYT1 kinases and positively regulated by CDC25C phosphatases. CHK1 inactivates CDC25C, activates WEE1, and promotes the degradation of CDC25. FAM122A is a negative regulator of the G2/M checkpoint and forms a complex with the phosphatase PP2A. When CHK1 phosphorylates FAM122A, PP2A is disinhibited and dephosphorylates WEE1, preventing its degradation and activating the G2/M checkpoint by WEE1. As single agents, ATR–CHK1–PKMYT1-WEE1 inhibitors are potent inhibitors of S phase and G2/M cell-cycle checkpoints leading to replication stress and early entry to mitosis. They also promote impairment of HRR during the S and G2 phases and induction of double-strand breaks.^86^

ATR-CHK1-WEE1 possesses different roles in replication fork stabilization. As mentioned, tumors with low expression of *SLFN11* and PARG downregulation depend on the ATR-CHK1-WEE1 axis to tolerate replication stress. *In vitro* and *in vivo* ATR-CHK1-WEE1 pathway inhibition re-sensitizes tumor cells to platinum and PARPi.^86^ For instance, ATR initiates DDR through several processes. This pathway recognizes replication stress and induces cell-cycle arrest to enable DNA repair.^87^ The combination of ATR inhibitor and PARPi has already been shown to reverse PARPi resistance in *in vitro* and *in vivo* models with different resistance mechanisms to PARPi.^88,89^ ATR inhibitors have shown promising efficacy in clinical trials. RP-3500 is a highly selective ATRi with clinical activity demonstrated in phase I/II TRESR study (NCT04497116) across a spectrum of tumor types and genomic alterations in DDR genes, including PARPi-resistant EOC with *BRCA1* or *RAD51C* mutations.^90^ Interestingly, the objective response rate (ORR) was 25% (5/20) among patients with EOC, 17 of whom presented with platinum-resistant disease and 18 of whom had prior PARPi treatment. The most common treatment-related adverse events (TRAEs) of all grades were anemia (81%), neutropenia (72%), and thrombocytopenia (45%).^90^

The synergy between ATRi and DNA-damaging agents has also been explored. Preliminary data from the phase II single-arm CAPRI trial (NCT03462342) showed clinical activity of ceralasertib and olaparib in PARPi-resistant EOC. The ORR was 46% (6/13 patients) in a heterogeneous group of women with platinum-sensitive relapsed EOC who had received a prior PARPi and progressed. Grade 3 toxicity occurred in 31% of patients, most commonly thrombocytopenia, anemia, and neutropenia.^91^ The addition of gemcitabine to the intravenous ATRi, berzosertib, demonstrated activity in phase II (NCT02595892) randomized study (berzosertib + gemcitabine *versus* gemcitabine alone) in platinum-resistant HGSOC. Median PFS favored the combination with 22.9 weeks for gemcitabine plus berzosertib *versus* 14.7 weeks for gemcitabine alone. Grades 3/4 TRAEs with the combination were neutropenia (47%) and thrombocytopenia (24%).^92^ Ongoing trials are evaluating the combination of ATRi with various chemotherapies (NCT04657068, NCT02264678, and NCT05147272).

WEE1 kinase, a G2 cell-cycle checkpoint regulator, promotes cell-cycle arrest and enhances apoptosis in the setting of DNA damage. The WEE-1 inhibitor (WEE1i), adavosertib, has demonstrated synergy with PARPi in preclinical studies.^93,94^ The randomized phase II non-comparative EFFORT trial (NCT03579316) showed the efficacy of adavosertib in PARPi-resistant EOC with an ORR of 23% with adavosertib alone and 29% in the combination of adavosertib plus olaparib arm. Frequent TRAEs included gastrointestinal effects (nausea, diarrhea), fatigue, and hematological toxicity.^95^ Similarly, in a phase I trial with another WEE1i, ZN-c3 (NCT04158336), the most frequent adverse events were nausea, vomiting, diarrhea, and fatigue. In that study, two of 16 patients had partial response.^96^ ZN-c3 is also being tested in combination with standard-of-care chemotherapy in platinum-resistant HGSOC in a phase Ib trial (NCT04516447). Preliminary results showed an ORR of 30.2% among 43 patients.^97^ More CDK1-selective than WEE1 inhibition is the PKMYT1 inhibition, which causes unscheduled activation of CDK1, leading to early mitosis.^98^ Evidence from preclinical studies has demonstrated that the PKMYT1 inhibitor RP-6306 has higher selectivity for cyclin E1-overexpressing cells both *in vitro* and *in vivo* and synergy with gemcitabine.^99^

Likewise, the synergy between PARPi and CDK4/6 inhibitors demonstrated in cancer cell lines derived from patients whose EOC shows high MYC expression and does not respond to PARPi.^100^ DNA polymerase Polθ is an enzyme encoded by *POLQ* important for MMEJ repair. Preclinical studies showed a synthetic lethal interaction between loss of the *POLQ* gene and deficiencies in genes that control DSBs repair and HR, including *BRCA1/2, ATM*, and *FANCD2*.^101,102^ Polθ is an emerging therapeutic target that confers synthetic lethality with defects in the 53BP1/Shieldin DNA repair complex. Two studies report specific Polθ inhibitors with *in vivo* efficacy, which could underpin a promising therapy to bypass PARPi resistance in HR-deficient tumors.^103,104^

Next-generation PARP1-selective inhibitors have entered phase I clinical trials. Current PARPis are equipotent against PARP1 and PARP2 enzymes. Animal data suggest that these drugs exert their therapeutic effects through PARP1, and toxicity comes mainly from the inhibition of PARP2.^105^ The initial data from the ongoing phase I/II PETRA trial (NCT04644068) of AZD5305, a highly selective PARP1 inhibitor, demonstrated favorable tolerability across different doses with no dose-limiting toxicities and preliminary signals of activity. The PETRA study included women with advanced EOC harboring a germline or somatic *BRCA1/2*, *PALB2*, or *RAD51C/D* mutation, who had received prior platinum and prior PARPi.^106^ AZD5305 is also being tested in combination with chemotherapy and antibody–drug conjugates (ADC) targeting HER2 or topoisomerase 1 (NCT04644068).

### Indirect inhibition of HR

Target oncoproteins such as EGFR, VEGF, MEK, and PI3K-AKT have been reported to impair HR indirectly.^50^ In a phase I trial combining olaparib with AKT inhibitor capicarsetib, 11 of 25 patients with EOC achieved clinical benefit, irrespective of *BRCA1/2* mutation.^107^ PARPi resistance is associated with the upregulation of the RAS/MAPK pathway. Therefore, MEK inhibition induced HR deficiency by decreasing *MRE11*, *RAD50*, *NBN*, and *BRCA1/2*. The ongoing phase I/II SOLAR trial (NCT03162627) combines MEK inhibitor selumetinib with olaparib and includes an expansion cohort of PARPi-resistant EOC. Preclinically, these two agents synergistically act to increase DNA damage and apoptosis in response to PARPi.^108^

Another way to inhibit HR indirectly is by targeting epigenetic regulators. Bromodomain containing 4 (BRD4) is a member of the BET protein family with roles in epigenetic gene regulation. BRD4 inhibitors have been shown to suppress HR-associated genes, including *CTIP*, *BRCA1, RAD51*, and *TOPBP1*, thereby generating a state of HR deficiency and synergy with PARPi.^109,110^ BET and PARP inhibition have demonstrated synergy in xenograft models with a reduction in tumor growth, increasing apoptosis, and DNA damage.^111^ Preclinically, BET inhibition has been shown to enhance antitumor immunity.^112^ A phase I/Ib trial is evaluating the safety and efficacy of ZEN-3694, a BET inhibitor, plus nivolumab with or without ipilimumab in solid tumors, including platinum-resistant EOC (NCT04840589).

### Modulation of the tumor microenvironment

The tumor microenvironment protects tumor cells by providing mechanical support or secreting a range of cytokines, enabling cancer to evade both the immune system and subsequent therapies. Cancer-associated fibroblasts (CAFs) participate in anticancer drug resistance by upregulating desmoplasia and pro-survival mechanisms within the tumor microenvironment.^113^ Tumor stroma is a complex component of the tumor microenvironment that contains numerous CAFs. Cytokine secretion, excess deposition, and aberrant remodeling of the extracellular matrix allow tumor cells to proliferate rapidly, develop resistance to therapy, and escape from immune surveillance.^77^ A high stromal cell ratio and extensive stromal desmoplasia have been reported as features of acquired chemo-resistance in the HGSOC.^54^ Strategies that modulate the immune microenvironment may overcome PARPi resistance in EOC.

Altered angiogenesis, desmoplastic stroma, and upregulation of drug efflux pump all impair drug delivery to tumor cells. ADC is a complex emerging therapy that consists of an antibody designed against a specific tumor cell target conjugated by a linker to a cytotoxic payload.^114^ Targeting tumor cell surface antigens and delivering the payload may be effective ways to overcome drug resistance. The single-arm phase III SORAYA trial evaluated Mirvetuximab soravtansine in 106 women with platinum-resistant HGSOC and high expression of folate receptor alpha. Patients were heavily pretreated, and 48% had received prior PARPi. The ORR was 32.4%, including five complete responses.^115^ In the post-PARP setting, the most critical factor in developing ADCs is to find relevant antigens to target in this population with a high affinity to ensure appropriate drug release into the cancer cell.

Antiangiogenic therapy induces a hypoxemic tumor microenvironment with the downregulation of HR genes and can potentially improve drug delivery. However, the phase IIb CONCERTO trial (NCT02889900) demonstrated low clinical activity of the cediranib–olaparib combination in heavily pretreated patients with *BRCA* wild-type and platinum-resistant EOC with ORR of 15.3% (9/59 patients).^116^ TRAEs were experienced by 40% of patients. The most common TRAEs were hypertension, fatigue, diarrhea, and nausea.^116^ The phase II EVOLVE trial assessed this combination of cediranib–olaparib in patients after progression on PARPi.^51^ Patients were enrolled into platinum-sensitive (*n* = 10), platinum-resistant (*n* = 10), or exploratory (*n* = 10) cohort of patients who had progressed on a PARPi and progressed again on subsequent standard chemotherapy, regardless of platinum sensitivity. The 16-week PFS was 55%, 50%, and 39%, respectively. The ORR was 20% in the platinum-resistant cohort as opposed to 0% in platinum-sensitive and 8% in exploratory cohorts. Grade 3 TRAEs were reported in 38% of patients, most commonly diarrhea and anemia.^51^

Translational analyses of EVOLVE with paired biopsies identified acquired mechanisms of PARP resistance, regardless of the platinum cohort. Patients with reversion mutations in *BRCA1/2* and other HR genes and *ABCB1* upregulation had worse outcomes than patients with intact *BRCA1/2* genes. The rate of cyclin E1 (*CCNE1*) amplification, a biomarker of platinum resistance, was higher in *BRCA1/2*-mutated than in *BRCA1/2* wild-type (33% *versus* 15%) tumors. This result was the opposite of prior reports^117^ and could be driven by heavily pretreated patients with partial active synthetic lethality. Downregulation of *SLFN11* was found in 7% of patients. The data suggest that patients with reversion mutations and *ABCB1* upregulation might not have benefited from the combination of cediranib and olaparib.^51^ Ongoing NIRVANA-R phase II trial is assessing niraparib and bevacizumab in patients with platinum-sensitive recurrence previously treated with PARPi (NCT04734665).

The combination of the DDR pathway and immune checkpoint inhibitors is an area of active investigation. PARPi propagates DNA damage and releases DNA fragments into the cytoplasm, activating the cyclic GMP-AMP synthase (cGAS)-stimulator of interferon genes (STING) signaling. This pathway recognizes extranuclear double-stranded DNA and triggers the IRF3-type I interferon pathway, which is a crucial mediator of the immune system and induces activation of different immune cell types.^118,119^ PARP inhibition has also been shown to inactivate GSK3β and increase the CD8^+^ T-cell infiltration.^120^ However, there is a debate if these pathways depending on the *BRCA* mutation status and may be compromised if the mechanism of PARP resistance is the restoration of HR proficiency.^121,122^ Another study with cell lines has demonstrated that the activation of the cGAS-STING pathway occurs either solely or more potently in *BRCA*-deficient tumors.^123^ However, data from the phase II trial (NCT02484404) combining olaparib and durvalumab did not support the preclinical findings of modulation of cGAS/STING pathway by PARPi. In this study, STING expression was not associated with clinical benefit.^124^

Other combinations of PARPi and immune checkpoint inhibitors in recurrent EOC were assessed in small studies and were generally well tolerated.^125^ The phase I/II basket trial MEDIOLA (NCT02734004) included one cohort of platinum-sensitive EOC with germline *BRCA1/2* mutation and previously treated with at least one platinum-based chemotherapy.^126^ The combination of durvalumab and olaparib demonstrated an ORR of 71.9% in this cohort. A second-stage phase II study was designed to test the addition of bevacizumab to olaparib and durvalumab in germline *BRCA* wild-type relapsed EOC patients.^127^ The triplet demonstrated superiority over the doublet for all the endpoints with an ORR of 87.1% (95% CI: 70.2–96.4) *versus* 34.4% (95% CI: 18.6–53.2).^127^ Recent data confirmed the efficacy with median OS of 26.1 months for olaparib plus durvalumab and 31.9 months for olaparib, durvalumab, and bevacizumab. The most common TRAEs were anemia and hypertension in patients who received the triplet combination.^128^

The phase I/II TOPACIO trial (NCT02657889) assessed the combination of pembrolizumab and niraparib^129^ in platinum-resistant EOC regardless of *BRCA* status. The ORR of 18% with the combination was reported; no significant differences were seen between patients with *BRCA1/2* mutation and wild-type tumors. TRAEs of at least grade 3 included anemia (11%), thrombocytopenia (9%), and hyperglycemia (4%).^129^ A phase II proof-of-concept trial assessing the combination of durvalumab and olaparib demonstrated an ORR of 14% (5/35 patients) in a predominantly platinum-resistant recurrent EOC. This study indicated immunomodulatory effects of olaparib/durvalumab in patients and that VEGF/VEGFR pathway blockade would improve the tumor responses.^124^ Recently, MOONSTONE/GOG-3032 phase II trial (NCT03955471) evaluated the combination of niraparib and dostarlimab in patients with platinum-resistant EOC without *BRCA* mutation who received prior bevacizumab. The ORR was 7.3% (3/41 patients), and the median PFS was 2.1 months. Futility was declared due to low ORR.^130^ Clinical trials in first-line evaluating the combination of a PARPi with antiangiogenics or programmed cell death protein 1/programmed cell death ligand 1 inhibitors are ongoing, and results are awaited (NCT03602859, NCT037401165, NCT03737643, NCT03522246).

## Biomarkers of resistance to PARPi

A wide range of mechanisms result in PARP resistance, and each individual with EOC may have more than one altered pathway. Furthermore, all mechanisms have the potential to evolve, and predicting PARPi resistance in the clinic is challenging. Platinum sensitivity strongly predicts response to PARPi, and their resistance mechanisms often overlap. There is no biological predictive biomarker of platinum resistance, and PFI is widely used as a clinical biomarker. However, this definition has been questioned and replaced by platinum treatment-free interval (TFIp), considered a continuous variable. HRD is a dynamic phenotype useful to predict response to platinum agents and PARPi.^131^ Clinically, HRD test results and PARPi responses can be discordant. This may be because tumors with reversion mutations remain with evidence of HRD on these tests or that an alternative HR-independent mechanism of resistance is prevailing.^132^ Therefore, methods to reliably determine the HRD status in HGSOC are of critical importance to stratify and optimize treatment. There are three main categories of HRD tests: HRR pathway-related genes, genomic scars or mutational signatures, and functional assays.^133^

Currently, the most common commercial tests are the myChoice CDx (Myriad Genetics) and Foundation Focus CDx BRCA LOH (Foundation Medicine). The myChoice CDx use next-generation sequencing to assess tumor Genomic Instability Score (GIS), which is a combination of copy number variations measures, including loss of heterozygosis (LOH), telomeric allelic imbalance, and large-scale state transitions. The Foundation Focus CDx *BRCA* LOH assesses large-scale LOH at the genomic level. Both assays include a *BRCA* mutation test. GIS is a continuous score that varies between 0 and 100. A tumor is considered ‘HRD positive’ when GIS ⩾ 42. The percentage of genomic LOH considered ‘HRD positive’ was 14% in the ARIEL2 trial^134^ and 16% in the ARIEL3 trial.^135^ Other non-commercial HRD tests are under development, such as the assay of KU Leuven and collaborators, which was recently presented in comparison with Myriad myChoice in patients from the PAOLA-1 trial. The Leuven HRD test showed a similar impact of olaparib on median PFS as Myriad myChoice test.^136^

Other integrative models based on mutational signatures have been developed. HRDetect^137^ and Classifier of Homologous Recombination Deficiency (CHORD)^138^ are HRD tests based on whole-genome sequencing (WGS) analysis. These tests reflect the accumulation of genomic mutational scars and correlate with HRD in *BRCA-*deficient tumors. CHORD was developed using WGS data of 3584 patients from a pan-cancer metastatic cohort, including EOC. CHORD uses a combination of 29 mutational features of three somatic mutation categories: single-base substitution, insertions and deletions, and structural variants.^138^ CHORD could detect HRD with overall low false-positive (<2%) and false-negative rates (<6%).^138^ HRDetect was first tested for breast cancer and combines HRD-induced point mutations and short indels with large-scale chromosomal alterations. HRDetect for EOC performed similarly well compared to HRD score by Myriad myChoice or Foundation Focus LOH tests.^139^ However, all tests that are based on assessing genomic scars have limitations and still miss many potential responders to DNA-damaging therapies or falsely predict a benefit from DNA-damaging therapies in situations where HR may have been restored.

Functional assays assessing current HRD status are under investigation and require validation before clinical use.^140^ Recently, the RECAP (Repair CAPacity) assay in breast cancer was published. The RECAP test is based on measuring *RAD51* foci formation in proliferating cells by immunofluorescence, and its concordance with HRDetect and CHORD was 70%.^141^ The basal *RAD51* foci score based on HR status, *BRCA1* promoter methylation, and the HRDetect score showed a correlation with PARPi activity in EOC PDXs. The expression level of RAD51 foci was strongly inversely correlated with olaparib responsiveness. In this study, the lower the foci score, the greater the sensitivity to olaparib.^142^ A newer functional RAD51 assay was preclinically validated *in vivo* in PDX models of EOC, triple-negative breast cancer, and prostate cancer. This immunofluorescence-based test detects RAD51 nuclear foci in formalin-fixed paraffin-embedded samples. Compared to HRR gene mutations and genomic HRD analysis, the RAD51 test showed higher accuracy of 67% for predicting PARPi response in HGSOC.^143^ Future studies are awaited to determine which test would be the most cost-effective and feasible within a clinically relevant timeframe to inform HRD status.

Considering all the mechanisms conferring resistance to PARP, identifying molecular characteristics at each progression might be essential in defining the subsequent most appropriate line of treatment. Early detection of resistant subclones by tumor biopsy sampling may be an option but is not easily applicable in clinical practice. Non-invasive methods assessing tumor genomics, such as cell-free DNA, circulating tumor cells, and exomes, are emerging.^144^ ctDNA may provide a picture of disease status, mechanisms of resistance developed and be used to monitor response. The *TP53* mutant allele fraction was detected in ctDNA in 18 patients treated with the rucaparib as part of phase II ARIEL2 trial to monitor treatment response. Detection of *TP53* mutation in ctDNA was performed by targeted amplicon deep sequencing to detect low-frequency mutations. Tumor tissue specimens were profiled using an NGS-based assay. Concordant *TP53* mutations were detected in tumor and ctDNA from the plasma of all 18 patients. Seven patients with >50% reduction of *TP53* in ctDNA at cycle 2 achieved a partial response.^145^ The advantage of *TP53* mutation to monitor response over other clinical tools, such as CA-125, has not yet been demonstrated. Some studies have demonstrated that *BRCA1/2* reversion mutations can be detected in circulating cell-free DNA.^146–148^ Other resistance mechanisms, such as restoration of HR or replication fork protection, might be more challenging to detect, as large genomic deletions are more difficult to detect in cfDNA. Yet, data in the maintenance setting remain scanty in ovarian cancer.

Identifying the compromised pathway or mutation conferring PARPi resistance may affect the clinical treatment decision. For example, the phase III ARIEL-4 trial showed significant improvement in median PFS with rucaparib compared to chemotherapy in women with *BRCA*-mutated, PARPi-naïve relapsed EOC. *BRCA* reversion mutations were assessed prior to trial therapy by ctDNA, and women with *BRCA*-mutated HGSOC were less likely to benefit from rucaparib, demonstrating resistance to PARPi, which could have been primary or acquired by exposure to two or more platinum regimens.^21^ In later lines, *CCNE1* amplification or overexpression is often found in platinum-resistant HGSOC and is one of the common PARPi acquired mechanisms of resistance.^51^ *CCNE1* amplification or overexpression increases replication stress, possibly resulting in vulnerability to WEE1 inhibition, for example. The phase II IGNITE trial evaluated the efficacy of adavosertib in women with recurrent platinum-resistant HGSOC with *cyclin E* overexpression with and without *CCNE1* gene amplification (defined as ≥8 copies). *Cyclin E* expression was assessed by immunohistochemistry and copy number by fluorescent in situ hybridization. A clinical benefit rate of 61% was demonstrated in the *a priori* defined biomarker-selected arm of *Cyclin E* overexpressed and non-amplified HGSOC.^149^

The development of drugs that target the DNA replication process is increasing the interest in biomarkers of replication stress. Konstatinopoulos and colleagues analyzed the replication stress biomarkers^150^ in patients from the phase II trial with gemcitabine alone or in combination with berzosertib (ATRi) (NCT02595892).^92^ Replication stress high tumors were defined as having at least one genomic replication stress alteration: loss of retinoblastoma pathway regulation (*CCNE1* amplification, *RB1* two-copy loss, *CDKN2A* two-copy loss), and/or oncogene-induced replication stress (*KRAS* amplification, *NF1* mutations, *ERBB2* amplification, *MYC* amplification, and *MYCL1* amplification). Patients with high replication stress tumors had prolonged PFS on gemcitabine monotherapy.^150^ Gemcitabine itself induces replication stress, and in cells already under stress, it provokes cell death.^151^ On the other hand, patients with low replication stress responded poorly to gemcitabine alone and benefited from the addition of the ATRi berzosertib,^150^ which may explain the synergism between them. Further investigations are needed to define replication stress and how to identify patients in the clinic.

## Conclusion

PARPi resistance is emerging as a common challenge in the clinic and will increase with the wide use of PARPi in the first-line setting, despite long treatment-free intervals and likely cure for more women. Exploiting additional DDR targets is a promising strategy for PARPi combination or sequential therapies. The primary issue will be the selection of patients, given the heterogeneity of both HGSOCs and PARPi mechanisms of resistance. Therefore, efforts must be made to identify and integrate biomarkers to target tumor molecular vulnerabilities. Biomarkers of PARPi resistance need to be validated in further studies, considering feasibility, cost, and applicability to clinical practice. Functional assays are under investigation and may provide more accurate HRD status and prediction of PARPi response. New therapeutic targets extending the concept of synthetic lethality provide an exciting area of research to overcome PARPi resistance. Nonetheless, much coordinated preclinical, clinical, and translational work will be necessary to bring these advances to clinical practice.

## Declarations

**Ethics approval and consent to participate:** Not applicable.

**Consent for publication:** Not applicable.

**Availability of data and materials:** Not applicable.
